# Timing and Length of Nocturnal Sleep and Daytime Napping and Associations With Obesity Types in High-, Middle-, and Low-Income Countries

**DOI:** 10.1001/jamanetworkopen.2021.13775

**Published:** 2021-06-30

**Authors:** Lap Ah Tse, Chuangshi Wang, Sumathy Rangarajan, Zhiguang Liu, Koon Teo, Afzalhussein Yusufali, Álvaro Avezum, Andreas Wielgosz, Annika Rosengren, Iolanthé M. Kruger, Jephat Chifamba, K. Burcu Tumerdem Calik, Karen Yeates, Katarzyna Zatońska, Khalid F. AlHabib, Khalid Yusoff, Manmeet Kaur, Noorhassim Ismail, Pamela Seron, Patricio Lopez-Jaramillo, Paul Poirier, Rajeev Gupta, Rasha Khatib, Roya Kelishadi, Scott A. Lear, Tarzia Choudhury, Viswanathan Mohan, Wei Li, Salim Yusuf

**Affiliations:** 1 Jockey Club School of Public Health and Primary Care, Chinese University of Hong Kong, Hong Kong, China; 2 Medical Research and Biometrics Center, State Key Laboratory of Cardiovascular Disease, Fuwai Hospital, National Center for Cardiovascular Diseases, Peking Union Medical College and Chinese Academy of Medical Sciences, Beijing, China; 3 Population Health Research Institute, McMaster University, Hamilton, Canada; 4 Division of Occupational and Environmental Health, Jockey Club School of Public Health and Primary Care, Chinese University of Hong Kong, Hong Kong, China; 5 Dubai Medical University, Hatta Hospital, Dubai Health Authority, Dubai, United Arab Emirates; 6 Research Division, Dante Pazzanese Institute of Cardiology, São Paulo, Brazil; 7 Department of Medicine, University of Ottawa, Ottawa, Canada; 8 Department of Molecular and Clinical Medicine, Sahlgrenska Academy, University of Gothenburg and Sahlgrenska University Hospital, Gothenburg, Sweden; 9 Africa Unit for Transdisciplinary Health Research, North-West University, Potcehfstroom, South Africa; 10 Department of Physiology, University of Zimbabwe College of Health Sciences, Harare, Zimbabwe; 11 Department of Health Management, Faculty of Health Sciences, Marmara University, Istanbul, Turkey; 12 Department of Medicine, Faculty of Health Sciences, Queen’s University, Kingston, Canada; 13 Department of Social Medicine, Wroclaw Medical University, Wroclaw Poland; 14 Department of Cardiac Sciences, King Fahad Cardiac Center, King Saud University College of Medicine, Riyadh, Saudi Arabia; 15 Universiti Teknologi MARA, Selayang, Malaysia; 16 UCSI University, Kuala Lumpur, Malaysia; 17 Department of Community Medicine and School of Public Health, Postgraduate Institute of Medical Education and Research, Chandigarh, India; 18 Department of Community Health, Faculty of Medicine, University Kebangsaan Malaysia, Kuala Lumpur, Malaysia; 19 Dpto Medicina Interna, Facultad de Medicina, Universidad de La Frontera, Temuco, Chile; 20 FOSCAL and Medical School, Universidad de Santander, Bucaramanga, Colombia; 21 Faculté de pharmacie, Université Laval, Québec, Canada; 22 Eternal Heart Care Centre and Research Institute, Jaipur, India; 23 Institute of Community and Public Health, Birzeit University, Birzeit, Palestine; 24 Isfahan Cardiovascular Research Center, Cardiovascular Research Institute, Isfahan University of Medical Sciences, Isfahan, Iran; 25 Faculty of Health Sciences, Simon Fraser University, Burnaby, Canada; 26 Independent University, Dhaka, Bangladesh; 27 Madras Diabetes Research Foundation, Dr Mohan’s Diabetes Specialities Centre, Chennai, India

## Abstract

**Importance:**

Obesity is a growing public health threat leading to serious health consequences. Late bedtime and sleep loss are common in modern society, but their associations with specific obesity types are not well characterized.

**Objective:**

To assess whether sleep timing and napping behavior are associated with increased obesity, independent of nocturnal sleep length.

**Design, Setting, and Participants:**

This large, multinational, population-based cross-sectional study used data of participants from 60 study centers in 26 countries with varying income levels as part of the Prospective Urban Rural Epidemiology study. Participants were aged 35 to 70 years and were mainly recruited during 2005 and 2009. Data analysis occurred from October 2020 through March 2021.

**Exposures:**

Sleep timing (ie, bedtime and wake-up time), nocturnal sleep duration, daytime napping.

**Main Outcomes and Measures:**

The primary outcomes were prevalence of obesity, specified as general obesity, defined as body mass index (BMI; calculated as weight in kilograms divided by height in meters squared) of 30 or greater, and abdominal obesity, defined as waist circumference greater than 102 cm for men or greater than 88 cm for women. Multilevel logistic regression models with random effects for study centers were performed to calculate adjusted odds ratios (AORs) and 95% CIs.

**Results:**

Overall, 136 652 participants (81 652 [59.8%] women; mean [SD] age, 51.0 [9.8] years) were included in analysis. A total of 27 195 participants (19.9%) had general obesity, and 37 024 participants (27.1%) had abdominal obesity. The mean (SD) nocturnal sleep duration was 7.8 (1.4) hours, and the median (interquartile range) midsleep time was 2:15 am (1:30 am-3:00 am). A total of 19 660 participants (14.4%) had late bedtime behavior (ie, midnight or later). Compared with bedtime between 8 pm and 10 pm, late bedtime was associated with general obesity (AOR, 1.20; 95% CI, 1.12-1.29) and abdominal obesity (AOR, 1.20; 95% CI, 1.12-1.28), particularly among participants who went to bed between 2 am and 6 am (general obesity: AOR, 1.35; 95% CI, 1.18-1.54; abdominal obesity: AOR, 1.38; 95% CI, 1.21-1.58). Short nocturnal sleep of less than 6 hours was associated with general obesity (eg, <5 hours: AOR, 1.27; 95% CI, 1.13-1.43), but longer napping was associated with higher abdominal obesity prevalence (eg, ≥1 hours: AOR, 1.39; 95% CI, 1.31-1.47). Neither going to bed during the day (ie, before 8pm) nor wake-up time was associated with obesity.

**Conclusions and Relevance:**

This cross-sectional study found that late nocturnal bedtime and short nocturnal sleep were associated with increased risk of obesity prevalence, while longer daytime napping did not reduce the risk but was associated with higher risk of abdominal obesity. Strategic weight control programs should also encourage earlier bedtime and avoid short nocturnal sleep to mitigate obesity epidemic.

## Key Points

**Question:**

Is late bedtime associated with general and abdominal obesity and does a heterogonous association exist between men and women?

**Findings:**

In this cross-sectional study of 136 652 participants from 26 countries from the Prospective Urban Rural Epidemiology study, after adjustment for a wide range of potential confounding factors, late nocturnal bedtime and short nocturnal sleep were associated with increased risk of general and abdominal obesity, while longer daytime napping could not compensate for the loss but further increased risk of abdominal obesity, especially among women.

**Meaning:**

These findings suggest that encouraging earlier bedtime and avoiding short nocturnal sleep may benefit weight control.

## Teaser

This cross-sectional study examines the associations of bedtime, sleep duration, and daytime napping with obesity in a multinational population.

## Introduction

Obesity is an evolving public health concern leading to serious health consequences of diabetes, cardiovascular diseases, cancer, and premature death.^1,2,3^ Globally, the prevalence of obesity in adults has doubled since 1980,^4^ which parallels decreased duration of sleep in modern society, possibly driven by high social and behavioral demands around the clock from work and increased modern technology keeping people awake at night.^5^ Sleep loss is becoming more common, with approximately one-third of adults sleeping less than 6 hours per night.^6^ Existing epidemiological studies widely demonstrated that chronic sleep loss behavior is associated with risk of weight gain and obesity,^7,8,9,10^ and the American Academy of Sleep Medicine and Sleep Research Society recommends that adults should sleep 7 or more hours per night regularly to promote optimal health, including healthy weight.^7^ However, there has been no guideline on the optimal timing to wake-up and go to sleep (ie, bedtime).

Increased exposure to light at night may make people go to bed late. Late bedtime behavior was found to be associated with obesity independent of sleep duration, but the findings are mixed.^11,12,13^ Emerging studies showed that napping during the daytime may reflect weakened circadian rhythm involved in the development of obesity, but the evidence from large studies is limited.^14,15^ Sex and age differences may exist for the association between bedtime and obesity, as women and elderly people are more likely to be classified as morning chronotypes than men and young people, yielding them more sensitive to weakened circadian rhythm induced by the delaying bedtime.^13,16^ However, in most of the previous studies among general adults, few large population-based studies specifically examined the independent association of sleep timing behavior from duration of nocturnal sleep and napping with the risk of specific obesity type. Additionally, potential sex differences and heterogeneities between younger and elderly people were less studied. Therefore, based on the Prospective Urban Rural Epidemiology (PURE) study of 136 652 adults from 26 countries, we aimed to assess the associations of specific obesity types with sleep timing (ie, bedtime, wake-up time) and daytime napping behavior after taking into account nocturnal sleep duration and a wide range of potential confounding factors; in addition, we aimed to examine whether there were heterogeneous associations between men and women and across age groups.

## Methods

This cross-sectional study was coordinated by the Population Health Research Institute (Hamilton, Ontario) in Canada and local ethics approval was obtained by all collaborating sites. Written informed consent was obtained from all participants. This report follows the Strengthening the Reporting of Observational Studies in Epidemiology (STROBE) reporting guideline for cross-sectional studies.

### Recruitment of Study Participants

The PURE study is a multinational community-based prospective urban-rural epidemiology study designed to quantify the global burden of cardiovascular diseases and risk factors in 26 countries involving 60 study centers, with 4 high-income countries (Canada, Sweden, Saudi Arabia, and United Arab Emirates), 17 middle-income countries (Ecuador, Argentina, Brazil, Chile, China, Colombia, Iran, Malaysia, Palestine, Poland, South Africa, Sudan, Philippines, Russia, Kazakhstan, Kyrgyzstan, and Turkey) and 5 low-income countries (Bangladesh, India, Pakistan, Tanzania, and Zimbabwe), classified according to the World Bank classification for 2006 when the study was initiated. Details of the study design and participant recruitment have been described previously.^17^ In brief, baseline data were collected at the community, household, and individual levels using standardized questionnaires, collecting information on sociodemographic factors, sleep, and other lifestyle behavior factors (eg, smoking, diet, and physical activity), past medical history, and family history of medical conditions.

Physical activity was assessed by total physical activities from occupational, transportation, housework, and leisure time according to the International Physical Activity Questionnaire,^18^ and categorized into low (<600 metabolic equivalent of task [MET]–min/wk; eg, having 3 days of walking for 30 min/d in 1 week), moderate (600-3000 MET-min/wk; eg, having 5 days of bicycling at a regular pace for 30 min/d in 1 week) and high (>3000 MET-min/week; eg, having 7 days of fast bicycling for 1 h/d in 1 week) activity. The total energy intake of each participant was estimated based on country-specific validated food frequency questionnaires.^19^ Among all 181 882 participants with anthropometric measurements, 45 230 were excluded from further data analyses owing to missing (because of either being enrolled prior to inclusion of sleep questions or no response) or implausible nocturnal sleep duration (or napping hours) or missing data of sex or age using the same inclusion and exclusion criteria of our previous report.^15^ Finally, a total of 136 652 men and women aged 35 to 70 years who completed baseline information on sleep and anthropometric measurements and recruited mainly during 2005 to 2009 were included. The flowchart of participant recruitment is shown in the eFigure in the Supplement.

### Sleep Timing, Nocturnal Sleep Duration, and Napping

Each participant’s usual sleep timing behaviors, mean nocturnal sleep duration, and daily napping behavior were obtained using the baseline standardized questionnaire. Sleep timing behaviors included bedtime and wake-up time, which were ascertained using the following question “During your longest or nocturnal sleep period, what time do you normally go to bed and wake up (in a 24-hour cycle)?” Daily habitual siesta or nap duration was also recorded. *Siesta* means a short afternoon rest or nap taken after the midday meal, which is a traditional lifestyle in some countries with particularly warm weather. Chronic short sleep was defined as mean nocturnal sleep duration of less than 6 hours,^20^ and chronic sleep deprivation was defined as mean nocturnal sleep duration of less than 5 hours.^21^ Bedtime was classified into 5 subgroups: 6 am to 8 pm, 8 pm to 10 pm, 10 pm to midnight, midnight to 2 am, and 2 am to 6 am, based on the human physiology of sleep and circadian rhythm of melatonin that peaks at 2 to 3 am, while keeping awake at night may suppress secretion of melatonin associated with an increased risk of obesity, cardiovascular diseases and cancers.^22,23^ Wake-up time was categorized into 3 subgroups: before 4 am, 4 am to 6 am, and after 6 am. For nocturnal sleep, we further defined late bedtime as midnight or later and daytime bedtime as between 6 am and 8 pm.

### Anthropometric Measurements

We measured each participant’s anthropometric data at the baseline survey. Trained health professionals recorded body height (in meters), body weight (in kilograms), and waist circumference (in centimeters) according to a standard protocol. Height was measured to the nearest 0.1 cm without shoes using a standard stadiometer, and weight was recorded in light clothing to the nearest 0.1 kg. Waist circumference was assessed using a flexible anthropometric tape to the nearest 0.1 cm at the midpoint between the lowest rib margin and anterior superior iliac crest immediately after expiration. Hip circumference was measured to the nearest 0.1 cm at the greatest protrusion of the gluteal muscles. Waist-to-hip ratio and waist-to-height ratio were calculated as the ratio of waist circumference to hip circumference and height, respectively. Body mass index (BMI) was calculated as weight in kilograms divided by height in meters squared.^2^

### Outcome Assessment

The main outcome was prevalence of obesity, which was further specified as general obesity and abdominal obesity. General obesity was defined as BMI of 30 or greater, according to the World Health Organization’s (WHO) definition and for international comparability. Abdominal obesity was defined as a waist circumference greater than 102 cm for men or greater than 88 cm for women, according to the Adult Treatment Panel III criteria.^24^

### Statistical Analysis

Baseline characteristics of participants were summarized and compared by subgroups of habitual bedtime. Continuous variables were expressed as mean with SD and compared by 1-way analysis of variance or Kruskal-Wallis test. Categorical variables were presented as frequency (percentage) and compared by 2-way χ^2^ test. To investigate the associations between sleep timing behaviors (ie, bedtime and wake-up time) and specific obesity types, multilevel logistic regression models were applied to calculate adjusted odds ratios (AORs) and the 95% CIs, using bedtime 8 pm to 10 pm or wake-up time 4 am to 6 am as the reference. Random effects for centers were used to account for the clustering within centers (which also accounts for country and region). The fully adjusted models for the association with bedtime included age, sex, education, location (urban or rural area), country income status (high, middle, or low), smoking status (current or former vs never smoker), drinking status (drinker vs nondrinker), family history of cardiovascular conditions (defined as father, mother, or siblings having diabetes, stroke, coronary heart disease, or high blood pressure), history of diabetes (defined as self-reported diabetes or using antidiabetic medications), depression, physical activity (expressed as MET-min/wk), nocturnal sleep duration (hours), total energy intake (kilocalories), and habitual naps (yes or no). We examined collinearity between potential confounding variables using variance inflation factors (VIFs), and all VIFs were less than 5, indicating a lower chance of collinearity problem between these covariables.

We examined the normality for the timing and length of nocturnal sleep, and they were not strictly normally distributed. We then classified nocturnal sleep duration into 7 subgroups (<5 hours, 5-6 hours, 6-7 hours, 7-8 hours, 8-9 hours, 9-10 hours, ≥10 hours) and napping duration into 3 subgroups (0, 0-1 hour, ≥1 hour) to examine the associations with the key outcomes by taking sleep duration of 7 or more to less than 8 hours and napping 0 hours as the reference. Additionally, we calculated midsleep time (MST) as a measurement of sleep phase, using noon as the reference and formula *bedtime* + (*nocturnal sleep duration* / 2), and treated MST as a continuous variable when examining the association with obesity types.

Given potential differences in the definition of abdominal obesity between ethnic groups, we performed sensitivity analyses by redefining ethnicity-specific abdominal obesity (ethnic groups were classified based on the self-reported ethnicity of the participants) according to the current recommended waist circumference thresholds for abdominal obesity prescribed by a Joint Interim Statement^25^; however, we only redefined general obesity in the sensitivity analyses for Chinese individuals according to the recommended criteria for Chinese population (ie, BMI≥28) because the WHO has no clear guideline for Asian populations.^26^ Missing data of sleep owing to no response (eFigure in the Supplement) were imputed using SAS statistical software Multiple Imputation Procedure (SAS Institute) to reanalyze the data as the sensitivity analysis. Subgroup analyses were performed according to categories of nocturnal short sleep (ie, <6 h/d vs ≥6 h/d), nocturnal sleep deprivation (ie, <5 h/d vs ≥5 h/d), and napping behavior (yes vs no) to examine if the associations of bedtime with obesity outcomes were modified by short sleep, sleep deprivation, or napping behavior. Consistency of associations of bedtime and MST with specific obesity types was also assessed across subgroups by introducing a multiplicative term in the full models for sex (men vs women), age (ie <65 years vs ≥65 years), physical activity (low, moderate, or high) and total energy intake (tertiles), and further stratified analysis was conducted only if the multiplicative interaction test was statistically significant. *P* values were 2-sided, and statistical significance was set at *P* < .05. All multivariate analyses were performed based on the complete data of 136 652 participants. Data analysis occurred from October 2020 through March 2021.

## Results

Among 136 652 people included analysis, 55 000 (40.2%) were men and 81 652 (59.8%) were women, with a mean (SD) age of 51.0 (9.8) years. Among the overall cohort, the mean (SD) nocturnal sleep duration was 7.8 (1.4) hours and 54 178 participants (39.7%) had daily napping behavior. A total of 19 660 participants (14.4%) had late bedtime behavior (ie, midnight or later), with a mean (SD) MST value of 15.8 (0.98) (ie, 3:48 am). The mean (SD) bedtime for people living in high-income countries was 10:54 pm (83 minutes), 33 minutes later than those in the middle-income countries (10:21 pm [81 minutes]) and 45 minutes later than those in the low-income countries (10:09 pm [75 minutes]). Urban residents went to bed later than people living in the rural areas (mean [SD] bedtime, 10:44 [80 minutes] pm vs 9:58 pm [77 minutes]). Median (interquartile range [IQR]) MST was later among people living in urban areas (2:30 am [2:00 am-3:00 am]) and middle-income countries (2:15 am [1:30 am-3:00 am]) or high-income countries (2:45 am (2:10 am-3:15 am]) compared with residents in rural areas (2:00 am [1:30 am-2:30 am]) and low-income countries (2:00 am [1:30 am-2:30 am]).

As shown in Table 1, compared with those who went to bed between 8 pm and 10 pm, individuals who had late bedtime were relatively younger, more often men and educated, and were more likely to live in urban areas and high-income countries. Moreover, people with late bedtime were more prone to late rise and had a relatively shorter nocturnal sleep duration and delayed MST, and they were more likely to be smokers and alcohol drinkers. These individuals consumed higher total energy intake but had less physical activity (Table 1). Compared with individuals who went to bed earlier, people who went to sleep later had higher mean (SD) BMI (eg, 25.2 [5.0] among people who went to bed from 8 pm-10 pm vs 28.9 [6.4] among those who went to bed from 2 am-6 am) and waist circumference (eg, 83.3 [12.6] cm among people who went to bed from 8 pm-10 pm vs 92.9 [14.9] cm among those who went to bed from 2 am-6 am) (Table 1). A similar trend was also observed for other anthropometric measurements, including hip circumference, waist-to-hip ratio and waist-to-height ratio (Table 1).

**Table 1.** Baseline Characteristics of Participants by Bedtime^a^

Characteristic	Bedtime, No. (%) (N = 136 652)				
	Daytime (n = 1769)^b^	8 pm-10 pm (n = 31 472)	10 pm-midnight (n = 83 751)	Midnight-2 am (n = 17 939)	2 am-6 am (n = 1721)
Age, mean (SD), y	52.5 (10.3)	51.7 (10.1)	51.1 (9.8)	49.8 (9.4)	49.2 (9.3)
Sex					
Men	727 (41.1)	12 055 (38.3)	33 868 (40.4)	7601 (42.4)	749 (43.5)
Women	1042 (58.9)	19 417 (61.7)	49 883 (59.6)	10 338 (57.6)	972 (56.5)
Education attainment					
None or primary school	1171 (66.5)	17 000 (54.1)	28 211 (33.7)	5918 (33.0)	536 (31.2)
Secondary, high, or higher secondary	459 (26.0)	11 372 (36.2)	32 366 (38.7)	6218 (34.7)	631 (36.7)
Trade, college, or university	132 (7.5)	3029 (9.6)	23 073 (27.6)	5790 (32.3)	552 (32.1)
Living location					
Urban	480 (27.1)	10 601 (33.7)	49 655 (59.3)	13 165 (73.4)	1380 (80.2)
Rural	1289 (72.9)	20 871 (66.3)	34 096 (40.7)	4774 (26.6)	341 (19.8)
Country income status					
High	83 (4.7)	1350 (4.3)	12 086 (14.4)	2443 (13.6)	444 (25.8)
Middle	1498 (84.7)	26 137 (83.0)	62 673 (74.8)	14 675 (81.8)	1168 (67.9)
Low	188 (10.6)	3985 (12.7)	8992 (10.7)	821 (4.6)	109 (6.3)
Smoking status					
No	1093 (62.1)	21 954 (70.5)	57 657 (69.1)	10 794 (60.3)	803 (46.7)
Current or former	666 (37.9)	9191 (29.5)	25 737 (30.9)	7120 (39.7)	915 (53.3)
Alcohol drinking					
No	973 (55.4)	21 583 (69.4)	54 413 (65.6)	11 328 (64.3)	984 (58.6)
Current or former	783 (44.6)	9524 (30.6)	28 565 (34.4)	6293 (35.7)	694 (41.4)
Physical activity, mean (SD), MET-min/wk	5886 (6338)	4815 (5555)	4428 (4891)	3891 (4628)	3568 (4738)
Total energy intake, mean (SD), kcal	2320 (1122)	2165 (975)	2207 (957)	2376 (1011)	2444 (1070)
Nocturnal sleep duration, mean (SD), h	9.7 (1.7)	8.8 (1.1)	7.6 (1.1)	6.7 (1.4)	6.3 (1.6)
Duration of daytime naps, mean (SD), h	1.0 (0.7)	1.0 (0.6)	1.0 (0.6)	1.1 (0.6)	1.2 (0.7)
Wake-up time, median (IQR), h:min in 24 h	5:30 (5:00-6:00)	6:00 (5:00-6:00)	6:00 (5:30-7:00)	7:00 (6:00-8:00)	9:00 (7:00-10:00)
MST, median (IQR), h:min in 24 h	NC	1:23 (1:00-1:31)	2:30 (2:00-2:45)	3:30 (3:00-4:00)	5:30 (4:45-6:15)
Anthropometric measurements, mean (SD)					
BMI	25.1 (5.4)	25.2 (5.0)	26.2 (5.0)	28.3 (5.7)	28.9 (6.4)
Waist circumference, cm	83.7 (13.4)	83.3 (12.6)	86.1 (13.2)	91.1 (13.8)	92.9 (14.9)
Hip circumference, cm	95.0 (11.6)	95.3 (10.7)	98.1 (10.8)	102.5 (11.6)	103.8 (12.9)
Waist-to-hip ratio	0.88 (0.09)	0.87 (0.08)	0.88 (0.08)	0.89 (0.09)	0.90 (0.09)
Waist-to-height ratio	0.53 (0.08)	0.52 (0.08)	0.53 (0.08)	0.56 (0.09)	0.57 (0.09)

Abbreviations: BMI, body mass index (calculated as weight in kilograms divided by height in meters squared); IQR, interquartile range; NC, not calculated; MET, metabolic equivalent of task; MST, midsleep time.

^a^ All variables were summarized and compared by subgroups of habitual bedtime and showed a *P* < .001. Continuous variables were compared by 1-way analysis of variance or Kruskal-Wallis test. Categorical variables were compared by χ^2^ test. All calculations were based on participants with complete data.

^b^ Defined as bedtime between 6 am and 8 pm. MST was not calculated for this group.

A total of 27 195 participants (19.9%) had general obesity and 37 024 participants (27.1%) had abdominal obesity. Compared with the bedtime 8 pm to 10 pm, late bedtime (ie, midnight-6am) was positively associated with prevalence of general obesity (AOR, 1.20; 95% CI, 1.12-1.29) and abdominal obesity (AOR, 1.20; 95% CI, 1.12-1.28). Participants with the latest bedtime (ie, 2 am-6 am) had the highest prevalence of general obesity (AOR, 1.35; 95% CI, 1.18-1.54) and abdominal obesity (AOR, 1.38; 95% CI, 1.21-1.58) (Table 2). Neither daytime bedtime (ie, 6am-8pm) nor wake-up time was significantly associated with obesity outcomes.

**Table 2.** Multilevel Logistic Regression Analyses for Associations of Sleep Timing With General Obesity and Abdominal Obesity

Sleep schedule	General obesity			Abdominal obesity		
	No (%)	AOR (95% CI)	*P* value	No (%)	AOR (95% CI)	*P* value
**Bedtime**						
Daytime^a^	292 (16.5)	0.98 (0.83-1.16)	.81	427 (24.1)	1.04 (0.90-1.20)	.59
8 pm-10 pm	4496 (14.3)	1 [Reference]	NA	6748 (21.4)	1 [Reference]	NA
10 pm-midnight	15 991 (19.1)	1.08 (1.03-1.14)	.004	22 125 (26.4)	1.06 (1.02-1.11)	.009
Midnight-2 am	5788 (32.3)	1.19 (1.11-1.28)	<.001	6959 (38.8)	1.19 (1.11-1.27)	<.001
2 am-6 am	628 (36.5)	1.35 (1.18-1.54)	<.001	765 (44.5)	1.38 (1.21-1.58)	<.001
**Wake-up time**						
Before 4 am	526 (20.6)	0.91 (0.79-1.04)	.17	720 (28.2)	0.89 (0.78-1.01)	.07
4 am-6 am	7085 (18.1)	1 [Reference]	NA	10 015 (25.5)	1 [Reference]	NA
After 6 am	19 584 (20.6)	1.04 (0.99-1.09)	.10	26 289 (27.7)	1.01 (0.97-1.06)	.52

Abbreviations: AOR, adjusted odds ratio; NA, not applicable.

^a^ Defined as bedtime between 6 am and 8 pm.

Compared with participants with nocturnal sleep duration of 7 to 8 hours, participants with sleep deprivation (ie, nocturnal sleep <5 h/d) had the highest prevalence of general obesity (AOR, 1.27; 95% CI, 1.13-1.43) and abdominal obesity (AOR, 1.16; 95% CI, 1.03-1.30) (Table 3). Nocturnal sleep duration longer than 6 hours was not associated with specific obesity types. However, longer daytime napping was significantly associated with increased prevalence of general obesity (eg, ≥1 hour: AOR, 1.22; 95% CI, 1.15-1.30) and abdominal obesity (eg, ≥1 hour: AOR, 1.39; 95% CI, 1.31-1.47) (Table 3). Moreover, an hourly delay of MST was found to be associated with significantly increased prevalence of general obesity (AOR, 1.05; 95% CI, 1.03-1.07) and abdominal obesity (AOR, 1.05; 95% CI, 1.03-1.07).

**Table 3.** Multilevel Logistic Regression Analyses for Associations of Nocturnal Sleep and Napping Duration With General Obesity and Abdominal Obesity

Measure	General obesity			Abdominal obesity		
	No (%)	AOR (95% CI)^a^	*P* value	No (%)	AOR (95% CI)^b^	*P* value
Nocturnal sleep duration, h						
<5	737 (28.6)	1.27 (1.13-1.43)	<.001	887 (34.4)	1.16 (1.03-1.30)	.01
5-6	1818 (28.3)	1.15 (1.07-1.25)	<.001	2212 (34.4)	1.07 (0.99-1.16)	.07
6-7	4141 (24.6)	1.05 (0.99-1.11)	.11	5319 (31.6)	1.05 (0.99-1.10)	.10
7-8	7152 (20.3)	1 [Reference]	NA	9665 (27.4)	1 [Reference]	NA
8-9	7124 (16.9)	0.95 (0.91-0.99)	.02	10 315 (24.5)	0.98 (0.94-1.02)	.27
9-10	3962 (17.3)	0.93 (0.88-0.99)	.02	5626 (24.5)	0.97 (0.92-1.02)	.23
>10	2261 (21.6)	0.96 (0.89-1.03)	.28	3000 (28.7)	0.95 (0.88-1.02)	.13
Nap duration, h						
0	16 574 (20.1)	1 [Reference]	NA	22 097 (26.8)	1 [Reference]	NA
0-1	8091 (19.0)	1.15 (1.11-1.20)	<.001	11 383 (26.7)	1.19 (1.15-1.23)	<.001
≥1	2530 (21.8)	1.22 (1.15-1.30)	<.001	3544 (30.5)	1.39 (1.31-1.47)	<.001

Abbreviations: AOR, adjusted odds ratio; NA, not applicable.

^a^ Adjusted for age, sex, education, location, country income status, smoking status, drinking status, family history of disease, diabetes, depression, physical activity, total energy intake, bedtime, napping, and center as random effect.

^b^ Adjusted for age, sex, education, location, country income status, smoking status, drinking status, family history of disease, diabetes, depression, physical activity, total energy intake, bedtime, nocturnal sleep duration, and center as random effect.

Further stratified multivariate analyses focused on the association of bedtime and MST with specific obesity types according to sex, age, lifestyle factors, length of nocturnal sleep, and napping behavior. A similar pattern of association was observed between the subgroups of interest with exceptions of sex and age. As shown in the Figure, the associations between bedtime and general or abdominal obesity among women were consistently higher than those among men (*P* for interaction < .001), except for the category daytime bedtime among men, in which an exceptional statistically significant AOR for abdominal obesity was observed. A significant positive association was found between MST and the general and abdominal obesity types, with a consistently higher AOR among women than that men and among people aged 65 years or older than those aged younger than 65 years (Table 4).

**Figure.**
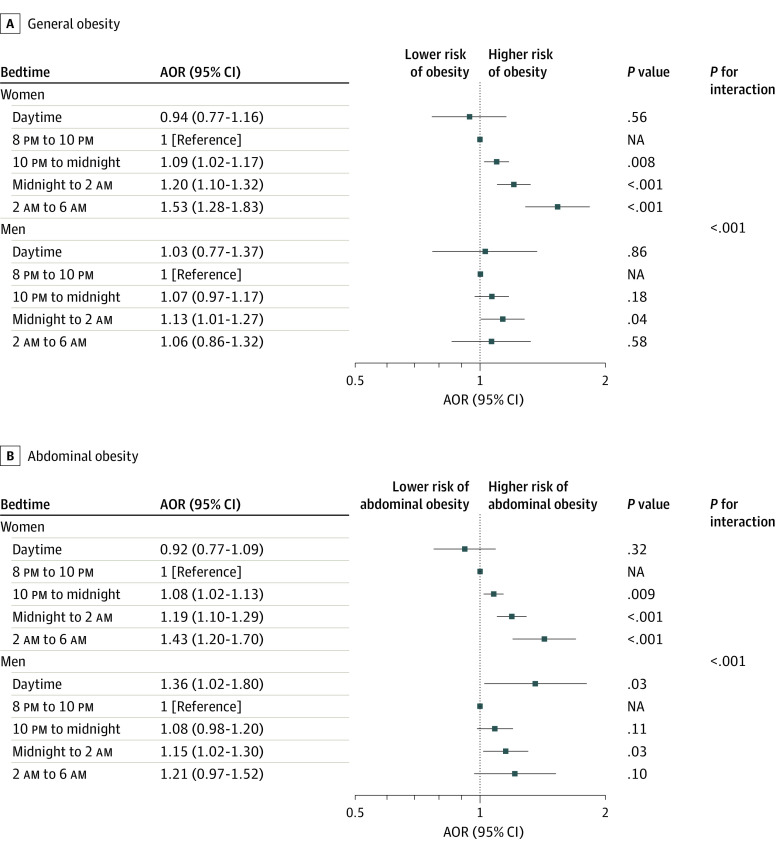
Associations Between Bedtime and Obesity, Stratified by Sex General obesity was defined as body mass index (calculated as weight in kilograms divided by height in meters squared) of 30 or greater, and abdominal obesity was defined as waist circumference greater than 102 cm in men or greater than 88 cm in women. AOR indicates adjusted odds ratio; NA, not applicable.

**Table 4.** Multilevel Logistic Regression Analyses for Associations of Mid-Sleep Time With Prevalent General Obesity and Abdominal Obesity in All Participants and by Sex or Age

Group	General obesity^a^		Abdominal obesity^b^	
	AOR (95% CI)	*P* value	AOR (95% CI)	*P* value
All participants^c^	1.05 (1.03-1.07)	<.001	1.05 (1.03-1.07)	<.001
Sex				
Men	1.01 (0.98-1.04)	.41	1.03 (1.00-1.07)	.03
Women	1.06 (1.03-1.08)	<.001	1.05 (1.03-1.07)	<.001
Age, y				
<65	1.04 (1.02-1.06)	<.001	1.03 (1.01-1.05)	<.001
≥65	1.04 (0.98-1.10)	.16	1.05 (1.00-1.11)	.05

Abbreviation: AOR, adjusted odds ratio.

^a^ Defined as body mass index (calculated as weight in kilograms divided by height in meters squared) of 30 or greater.

^b^ Defined as waist circumference as greater than 102 cm in men or greater than 88 cm in women.

^c^ Adjusted for age, sex, education, location, country income status, smoking status, drinking status, family history of disease, diabetes, depression, physical activity, total energy intake, nocturnal sleep duration, naps, and center as random effect.

In sensitivity analysis, replacing the cutoff points of general obesity and abdominal obesity using the criteria for specific ethnic groups did not change the patterns of associations with specific obesity types, although the association with wake-up time became statistically significant (eTable 1 in the Supplement). The associations remained almost unchanged after multiple imputation of missing sleep data (eTable 2 in the Supplement).

## Discussion

This large multinational population-based cross-sectional study of participants from 26 countries in the PURE study found that people from urban areas and high-income countries were more likely to have later bedtimes. Late bedtime behavior was associated with higher levels of anthropometric measurements of obesity and prevalence of general obesity or abdominal obesity, particularly in women. Nocturnal sleep shorter than 5 hours per night was significantly associated with obesity, especially general obesity; furthermore, longer daytime napping could not compensate for the loss of nocturnal sleep but may further increase the potential risk, particularly for abdominal obesity. Nocturnal sleep of 6 hours or longer was not associated with increased prevalence of any obesity type.

Current sleep consensus statements, such as those from the American Academy of Sleep Medicine and Sleep Research Society,^7^ recommend that adults should sleep at least 7 hours per night on a regular basis to promote optimal health. Our large multinational study including 136 652 participants from 26 countries found that the positive association of sleep duration with general or abdominal obesity was restricted to short sleep duration of less than 6 hours per night. Our study provides additional evidence for recommendation updates for the optimal nocturnal sleep duration from the currently suggested minimum of 7 hours to a minimum of 6 hours per night to promote healthy weight for adults. To our knowledge, there has been no recommendation on sleep timing (ie, time to bed and time to wake up) for health and well being. Recent research among children and adolescents has suggested that later bedtime may increase BMI or obesity risk,^27,28,29^ but findings of studies among adults are conflicting.^13,14,30,31,32^ In the Osteoporotic Fractures in Men and Study of Osteoporotic Fractures study by Patel et al^14^ involving 3053 older men and 2985 older women, higher BMI was found among those who were the latest to bed, particularly in women. A 2018 study from Japan^13^ of 9289 adults found that self-reported late bedtime was associated with general obesity among older adults after adjustment of sleep duration, but physical activity and total energy intake were not addressed. However, conflicting findings were reported in another survey among 13 429 US Hispanics/Latino individuals by Knutson et al,^31^ in which self-reported late bedtime was associated with lower BMI among adults younger than 36 years. None of these studies attempted to specifically explore the association of sleep with abdominal obesity, probably because these studies were relatively small and thus had difficulties in disentangling the role of specific bedtime from complex obesogenic factors and sleep duration owing to limited power. Our study with a larger sample size investigated the associations of specific bedtime with specific obesity types by addressing nocturnal sleep duration, napping, and a wide range of confounding factors and found that later bedtime was associated with higher risk of general or abdominal obesity. Our findings are reliable, as shown by the consistent trend for the association between bedtime and obesity outcomes after the WHO’s obesity criteria were replaced by ethnicity-specific obesity criteria in the sensitivity analysis.

Bedtime is a distinct determinant of sleep behavior that may impact obesity risk, possibly through affecting circadian rhythms. Human endogenous circadian rhythms are coordinated by the suprachiasmatic nucleus of the hypothalamus, with a day/night cycle of approximate 24 hours, which is synchronized to the environmental clues stimulated by the cycles of activity/rest, sleep/wake, and/or fasting/feeding.^22^ Delaying bedtime and delayed sleep phase may induce a greater risk for circadian misalignment, which has been proposed to be the underlying cause for abdominal obesity in particular in studies among individuals who perform nightshift work.^23,33^ Late bedtime or delayed sleep phase may be associated with more exposure to light at night, causing prolonged suppression of melatonin secretion from the pineal gland and leading to a weakened or misaligned circadian rhythm.^22^ Misalignment of circadian rhythm has been associated with decreased levels of leptin but increased levels of plasma glucose, corticosteroids, and systemic inflammation that are associated with cardiometabolic abnormalities, and women are more susceptible than men.^21,34,35,36,37^ These biological mechanisms may explain the observed higher risk of obesity among women with later bedtime or delayed sleep phase than that of men in our study, consistent with our hypothesis that women were more susceptible to negative outcomes associated with late bedtime behavior than men. The exceptionally higher association of bedtime by day with abdominal obesity among men may be explained by their engagement in nightshift work; however, this issue remains unresolved in our study, since we did not collect occupational history or shift work schedules.

Associations between wake-up time and obesity among adolescents and adults have been unclear. In our multinational population-based study, there were no associations between waking up later than 6 am and obesity outcomes, except in the sensitivity analysis, in which the WHO’s obesity criteria were replaced by ethnicity-specific obesity criteria, suggesting that some uncertainties may exist, and this would need to be further investigated in future studies. On the other hand, while daytime napping may be associated with considerable benefits in terms of mood, alertness, and cognitive performance,^38^ napping could be a practical response to daytime sleepiness resulting from sleep loss, as late bedtime and early wake-up time could cause circadian misalignment, resulting in a prolonged high level of circulating melatonin after waking up, which, in turn, leads to longer napping as replacement. Circadian misalignment elevates cortisol level, which favors visceral fat deposition more than the peripheral fat,^39^ and this may explain higher abdominal obesity prevalence among our study participants who took longer napping periods.

### Limitations

Our study has a few limitations. First, because this is a cross-sectional study, we are limited to only detecting associations and not causal directions. While we hypothesized that sleep shortness increases risk for obesity, there are situations in which obesity can lead to disruptive sleep, such as sleep apnea. Only baseline results were included, since we did record anthropometric data systematically during follow-up. We expect to record this in the next round of follow-up and thereby assess whether these findings can be confirmed prospectively. Second, recall bias may impact sleep timing and sleep duration, since these were self-reported; however, if recall bias is present, this is likely a nondifferential misclassification, which would lead to an underestimation of the risk estimate. Third, we did not record information on sleep quality, which may affect obesity outcomes. Sleep quality may be affected by comorbid conditions (eg, depression, chronic cardiovascular or respiratory diseases), but we adjusted for comorbid conditions in the multivariate analysis; therefore, the confounding effect of sleep quality in our study is likely to be minimized. Also, previous studies have reported that sleep quality was not an independent risk factor for increased BMI after sleep duration was considered.^27,40^ Forth, missingness of sleep data may be a concern. We performed sensitivity analyses using a multiple imputation approach, and the results were comparable with those not using imputation. Additionally, we did not examine the role of chronotype (expressed as the propensity of an individual to rest or be active within a 24-hour cycle) and late night activities (eg, night-time eating behavior, nightshift work)^23,27,41^ that may contribute to the association between late bedtime and obesity, and this area of research is of important public health implication deserving further research.

## Conclusions

This cross-sectional study provided consistent epidemiological evidence from multinational adult populations that late nocturnal bedtime and delayed sleep phase may be independent risk factors for general obesity and abdominal obesity. Short nocturnal sleep less than 6 hours was positively associated with obesity; daytime napping could not revert the risk, but it may leave individuals more susceptible to abdominal obesity. Our findings suggest that strategic weight control programs should also encourage earlier bedtime and avoidance of short nocturnal sleep to mitigate obesity risk. Moreover, further prospective studies with objective exposure assessment on sleep pattern with longitudinal measurements on specific obesity types would be worthwhile.

## Notes

Supplement. **eFigure.** Flowchart of Recruitment of Baseline Participants **eTable 1.** Sensitivity Analysis for Sleep Timing, Duration of Nocturnal Sleep and Napping by Using Ethnicity-Specific Criteria to Measure Obesity Outcomes **eTable 2.** Multilevel Logistic Regression Analyses for Sleep Timing, Duration and Naps After Multiple Imputation **eAppendix.** Prospective Urban Rural Epidemiology (PURE) Study Group
