# Error in Funding/Support

**DOI:** 10.1001/jamanetworkopen.2021.24602

**Published:** 2021-08-04

In the Original Investigation titled “Timing and Length of Nocturnal Sleep and Daytime Napping and Associations With Obesity Types in High-, Middle-, and Low-Income Countries,”^1^ published June 30, 2021, there was an error in the Saudi Arabia section of the Funding/Support. This article has been corrected.^1^
