# Supplementary material for: Timing and Length of Nocturnal Sleep and Daytime Napping and Associations With Obesity Types in High-, Middle-, and Low-Income Countries
Source: JAMA Netw Open. 2021 Jun 30;4(6):e2113775. doi: 10.1001/jamanetworkopen.2021.13775 (PMC8246307; doi:10.1001/jamanetworkopen.2021.13775)
Supplement: Supplement. — eFigure. Flowchart of Recruitment of Baseline Participants eTable 1. Sensitivity Analysis for Sleep Timing, Duration of Nocturnal Sleep and Napping by Using Ethnicity-Specific Criteria to Measure Obesity Outcomes eTable 2. Multilevel Logistic Regression Analyses for Sleep Timing, Duration and Naps After Multiple Imputation eAppendix. Prospective Urban Rural Epidemiology (PURE) Study Group [file jamanetwopen-e2113775-s001.pdf]

Supplemental Online Content

Tse LA, Wang C, Rangarajan S, et al. Timing and length of nocturnal sleep and daytime napping and associations with obesity types in high-, middle-, and low-income countries. *JAMA Netw Open*. 2021;4(6):2113775. doi:10.1001/jamanetworkopen.2021.13775

eFigure. Flowchart of Recruitment of Baseline Participants

eTable 1. Sensitivity Analysis for Sleep Timing, Duration of Nocturnal Sleep and Napping by Using Ethnicity-Specific Criteria to Measure Obesity Outcomes

eTable 2. Multilevel Logistic Regression Analyses for Sleep Timing, Duration and Naps After Multiple Imputation

eAppendix. Prospective Urban Rural Epidemiology (PURE) Study Group

This supplemental material has been provided by the authors to give readers additional information about their work.

© 2021 Tse LA et al. *JAMA Network Open.*

eFigure. Flowchart of Recruitment of Baseline Participants

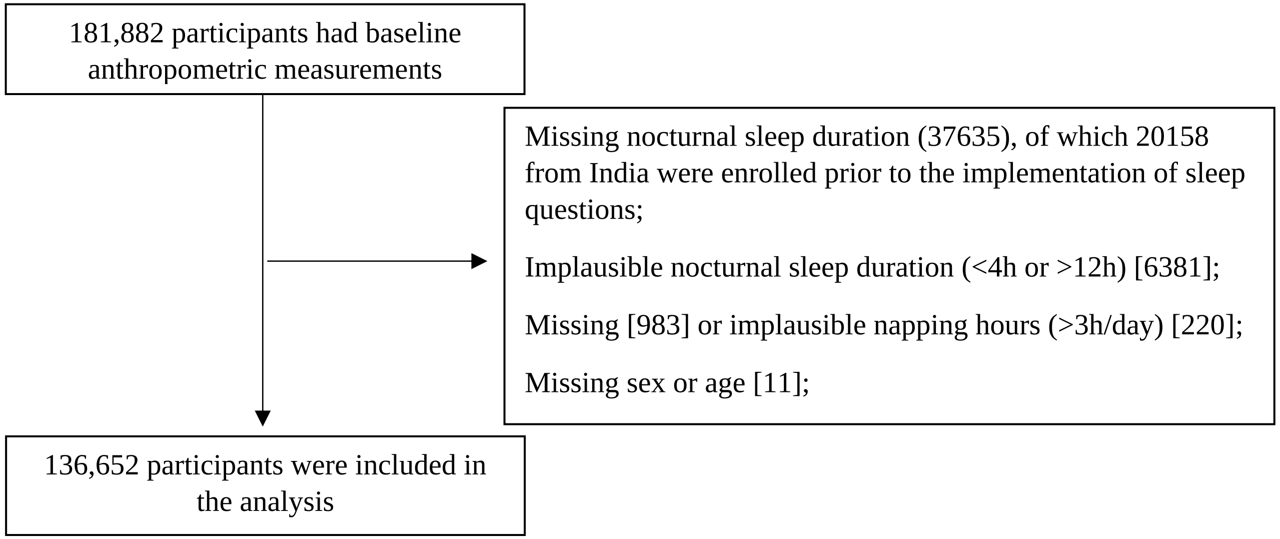

© 2021 Tse LA et al. *JAMA Network Open.*

eTable 1. Sensitivity Analysis for Sleep Timing, Duration of Nocturnal Sleep and Napping by Using Ethnicity-Specific Criteria to Measure Obesity Outcomes[a]

	**General obesity** [b]			**Abdominal obesity** [b]		
**Sleep patterns**	**No (%)**	**AOR (95% CI)**	**P value**	**No (%)**	**AOR (95%**	**P value**
Bedtime						
Daytime [c]	328 (18.5)	1.00 (0.86, 1.17)	0.98	771 (43.6)	0.93 (0.82, 1.05)	0.25
8PM – 10PM	5687 (18.1)	1.0		13631 (43.3)	1.0	
10PM – 00AM	18622 (22.2)	**1.08 (1.03, 1.14)**	**<0.001**	42701 (51.0)	**1.08 (1.04, 1.12)**	**<0.001**
00AM-2AM	5951 (33.2)	**1.20 (1.13, 1.29)**	**<0.001**	11379 (63.4)	**1.21 (1.14, 1.28)**	**<0.001**
2AM – 6AM	643 (37.4)	**1.37 (1.20, 1.56)**	**<0.001**	1127 (65.5)	**1.21 (1.06, 1.39)**	**0.005**
Wake-up time						
Before 4AM	542 (21.3)	0.92 (0.81, 1.06)	0.26	1338 (52.5)	0.81 (0.72, 0.90)	<0.001
4AM-6AM	8120 (20.7)	1.0		19468 (49.6)	1.0	
After 6AM	22569 (23.8)	**1.05 (1.01, 1.09)**	**0.03**	48803 (51.5)	**1.06 (1.03, 1.10)**	**<0.001**
Nocturnal sleep duration						
<5 hours	746 (28.9)	**1.25 (1.11, 1.41)**	**<0.001**	1554 (60.3)	1.08 (0.97, 1.21)	0.16
5-6 hours	1851 (28.8)	**1.14 (1.05, 1.23)**	**0.001**	3884 (60.4)	**1.09 (1.02, 1.18)**	**0.01**
6-7 hours	4369 (25.9)	1.04 (0.99, 1.10)	0.11	9560 (56.8)	1.04 (0.99, 1.09)	0.12
7-8 hours	8136 (23.1)	1.0		18521 (52.5)	1.0	
8-9 hours	8781 (20.8)	0.95 (0.91, 0.99)	0.02	20341 (48.3)	0.96 (0.93, 1.00)	0.04
9-10 hours	4843 (21.1)	0.95 (0.90, 1.00)	0.04	10577 (46.1)	0.92 (0.88, 0.96)	<0.001
≥10 hours	2505 (23.9)	0.96 (0.90, 1.03)	0.30	5172 (49.4)	0.95 (0.90, 1.02)	0.14

© 2021 Tse LA et al. *JAMA Network Open.*

Nap duration (hour)						
0h	18520 (22.5)	1.0		41200 (50.0)	1.0	
0-1h	9736 (22.9)	**1.14 (1.10, 1.18)**	**<0.001**	22088 (51.9)	**1.18 (1.15, 1.22)**	**<0.001**
>1h	2975 (25.6)	**1.20 (1.13, 1.27)**	**<0.001**	6321 (54.4)	**1.26 (1.20, 1.32)**	**<0.001**

As for bedtime and wake-up time, adjusted for age, sex, education, location, country income status, smoking, drinking, family history of disease, diabetes, probable depression, physical activity, total energy intake, nocturnal sleep duration, naps and center as random effects;

As for nocturnal sleep duration, adjusted for age, sex, education, location, country income status, smoking, drinking, family history of disease, diabetes, probable depression, physical activity, total energy intake, bedtime, napping and center as random effects;

As for napping, adjusted for age, sex, education, location, country income status, smoking, drinking, family history of disease, diabetes, probable depression, physical activity, total energy intake, bedtime, nocturnal sleep duration and center as random effects.

[a] Analysis population are those with baseline physical measurements (136,652)

[b] Keeping using WHO's criteria for other ethnicities but adopting Chinese obesity criteria to measure general obesity for Chinese population (≥28kg/m^2); using IDF's ethnicity-criteria to measure abdominal obesity (WC>90cm or >94cm for male; WC>80cm for female).

[c] Referring to bedtime between 6AM and 8PM.

© 2021 Tse LA et al. *JAMA Network Open.*

eTable 2. Multilevel Logistic Regression Analyses for Sleep Timing, Duration and Naps After Multiple Imputation

	**General obesity [b]**		**Abdominal obesity [b]**	
**Sleep schedule/duration**	**AOR (95% CI)**	**P value**	**AOR (95% CI)**	**P value**
Bedtime				
Daytime [a]	**1.17 (1.10 - 1.26)**	**<0.001**	**1.15 (1.08 - 1.22)**	**<0.001**
8PM – 10PM	1.0		1.0	
10PM – 00AM	**1.07 (1.03 - 1.12)**	**0.002**	**1.07 (1.03 - 1.12)**	**<0.001**
00AM-2AM	**1.21 (1.14 - 1.29)**	**<0.001**	**1.21 (1.14 - 1.28)**	**<0.001**
2AM – 6AM	**1.29 (1.16 - 1.44)**	**<0.001**	**1.41 (1.26 - 1.59)**	**<0.001**
Wake-up time				
Before 4AM	**0.89 (0.82 – 0.97)**	**0.008**	**0.88 (0.81 – 0.96)**	**0.003**
4AM-6AM	1.0		1.0	
After 6AM	1.03 (0.99 - 1.07)	0.17	1.03 (0.98 - 1.07)	0.19
Nocturnal sleep duration				
<5 hours	**1.19 (1.10 - 1.29)**	**<0.001**	**1.14 (1.06 - 1.22)**	**<0.001**
5-6 hours	**1.11 (1.04 - 1.18)**	**0.00**	1.06 (0.99 - 1.13)	0.09
6-7 hours	1.04 (0.99 - 1.09)	0.08	1.04 (1.00 - 1.09)	0.06
7-8 hours	1.0		1.0	
8-9 hours	0.95 (0.91 - 0.99)	0.01	0.98 (0.94 - 1.02)	0.32
9-10 hours	0.96 (0.92 - 1.01)	0.12	0.99 (0.95 - 1.04)	0.67
>10 hours	0.98 (0.92 - 1.04)	0.52	1.00 (0.94 - 1.05)	0.87
Nap duration (hour)				
0	**1.0**		**1.0**	
0-1	**1.12 (1.09 - 1.16)**	**<0.001**	**1.15 (1.12 - 1.19)**	**<0.001**
≥1	**1.15 (1.09 - 1.21)**	**<0.001**	**1.29 (1.23 - 1.35)**	**<0.001**

Analysis population are those with baseline physical measurements after multiple imputation of missing data of sleep (161,724).

Sleep timing: Adjusted for age, sex, education, location, country income status, smoking, drinking, family history of disease, diabetes, depression, physical activity, total energy intake, nocturnal sleep duration, naps, and center as random effect.

Nocturnal sleep duration: Adjusted for age, sex, education, location, country income status, smoking, drinking, family history of disease, diabetes, depression, physical activity, total energy intake, bedtime, naps, and center as random effect.

Nap duration: Adjusted for age, sex, education, location, country income status, smoking, drinking, family history of disease, diabetes, depression, physical activity, total energy intake, nocturnal sleep duration, bedtime, and center as random effect.

[a] Referring to bedtime between 6AM and 8PM.

[b] Keeping using WHO's criteria for other ethnicities but adopting Chinese obesity criteria to measure general obesity for Chinese population (⩾28kg/m2); using IDF's ethnicity-criteria to measure abdominal obesity (WC>90cm or >94cm for male; WC>80cm for female).

© 2021 Tse LA et al. *JAMA Network Open.*

eAppendix. Prospective Urban Rural Epidemiology (PURE) Study Group

PURE Project Office Staff, National Coordinators, Investigators, and Key Staff: Project office (Population Health Research Institute, Hamilton Health Sciences and McMaster University, Hamilton, Canada): S. Yusuf* (principal investigator).
S. Rangarajan (Program Manager Manager); K. K. Teo, S. S. Anand, C. K. Chow, M. O'Donnell, A. Mente, D. Leong, A. Smyth, P. Joseph, M. Duong, O. Kurmi, R. D'Souza, M. Walli-Attaei, B. Balaji, R. Naito, S. Islam (Statistician), W. Hu (Statistician), C. Ramasundarahettige (Statistician), P. Sheridan (Statistician), S. Bangdiwala, L. Dyal, A. Casanova, M. Dehghan (Nutrition Epidemiologist), G. Lewis (Associate Program Manager), D. Agapay, A. Aliberti, N. Aoucheva, A. Arshad, A. Reyes, B. Bideri, R. Buthool, S. Chin, M. Di Marino, R. Frances, S. Gopal, M. Jakymyshn, N. Kandy, I. Kay, J. Lindeman, G. McAlpine, E. McNeice, M(a). Mushtaha, M(o). Mushtaha, R. Patel, D. Pattath, S. Ramacham, E. Ramezani, J. Rimac, F. Shifaly, J. Swallow, M. Trottier, S. Trottier, R. Solano, A. Zaki, B. Zhang, V. Zhang
Core Laboratories: M. McQueen, S. Lamers, J. Keys (Hamilton), X. Wang (Beijing, China), A. Devanath (Bangalore, India).
Argentina: R. Diaz*, A. Orlandini, P. Lamelas, M. L. Diaz, A. Pascual, M. Salvador, C. Chacon; **Bangladesh:** O. Rahman*, R. Yusuf*, S. A. K. S. Ahmed, T. Choudhury, M. Sintaha, A. Khan, O. Alam, N,. Nayeem, S. N. Mitra, S. Islam, F. Pasha; **Brazil:** A. Avezum*, C. S. Marcilio, A. C. Mattos, G. B. Oliveira; **Canada:** K. Teo*, S. Yusuf*, Sumathy Rangarajan, A. Arshad, B. Bideri, I. Kay, J. Rimac, R. Buthool, S. Trottier, G. Dagenais, P. Poirier, G. Turbide, D. Auger, A. LeBlanc De Bluts, M. C. Proulx, M. Cayer, N. Bonneville, S. Lear, V. de Jong, A. N. Saidy, V. Kandola, E. Corber, I. Vukmirovich, D. Gasevic, A. Wielgosz, A. Pipe, A. Lefebvre, A. Pepe, A. Auclair, A. Prémont, A. S. Bourlaud; **Chile:** F. Lanas*, P. Serón, M. J. Oliveros, F. Cazor, Y. Palacios; **China:** Liu Lisheng*, Li Wei*, Chen Chunming#, Zhao Wenhua, Hu Bo, Yin Lu, Zhu Jun, Sun Yi, Wang Yang, Deng Qing, Jia Xuan, Zhang Hongye, Bo Jian, Liang Yan, He Xinye, Xie Liya, Liu Zhiguang, Yao Chenrui, Wang Xingyu, Liu Xu, Gao Nan, Bai Xiulin, Cheng Xiaoru, Wang Chuangshi, Li Sidong, Liu Weida, Lang Xinyue, Liu Xiaoyun, Zhu Yibing, Ren Yingjuan, Dai Xi, Wang Liping, Han Guoliang, Song Rui, Cao Zhuangni, Sun Yaya, Li Xiangrong, Wang Jing, Wang Li, Peng Ya, Li Xiaoqing, Li Ling, Wang Jia, Zou Jianmei, Gao Fan, Tian Shaofang, Li Yongmei, Bi Yanhui, Li Xin, Zhang Anran, Wu Dandan, Cheng ying, Xiao Yize, Lu Fanghong, Li Yindong, Hou Yan, Zhang Liangqing, Guo Baoxia, Liao Xiaoyang, Chen Di, Zhang Peng, Li Ning, Ma Xiaolan, Lei Rensheng, Fu Minfan, Liu Yu, Xing Xiaojie, Yang Youzhu, Zhao Shenghu, Xiang Quanyong, Tang Jinhua, Liu Zhengrong, Qiang Deren, Li Xiaoxia, Xu Zhengting, Aideeraili. Ayoupu, Zhao Qian; **Colombia:** P. Lopez-Jaramillo*, P. A. Camacho-Lopez, J. Otero-Wandurraga, D. I. Molina, C. Cure-Cure, M. Perez, E. Hernandez, E. Arcos, C. Narvaez, A. Sotomayor, H. Garcia, G. Sanchez, F. Cotes, A. Rico, M. Duran, C. Torres; **India:** P. Mony*, M. Vaz*, S. Swaminathan, K. Shankar, A. V. Kurpad, K. G. Jayachitra, N. Kumar, H. A. L. Hospital, V. Mohan*, M. Deepa, K. Parthiban, M. Anitha, S. Hemavathy, T. Rahulashankiruthiyayan, D. Anitha, R. M. Anjana, R. Dhanasekar and S. Sureshkumar, D. Anitha, K. Sridevi, R. Gupta*, R. B. Panwar, I. Mohan, P. Rastogi, S. Rastogi, R. Bhargava, R. Kumar, J. S. Thakur, B. Patro, R. Mahajan, P. Chaudary, V. Raman Kutty, K. Vijayakumar*, K. Ajayan, G. Rajasree, A. R. Renjini, A. Deepu, B. Sandhya, S. Asha, H. S. Soumya, R. Kumar*, M. Kaur, P. V.

© 2021 Tse LA et al. *JAMA Network Open.*

M. Lakshmi, V. Sagar; **Iran:** R. Kelishadi*, A. Bahonar, N. Mohammadifard, H. Heidari; **Kazakhstan:** K. Davletov*, B. Assembekov, B. Amirov; **Kyrgyzstan:** E. Mirrakhimov*, S. Abilova, U. Zakirov, U. Toktomamatov; **Malaysia:** K. Yusoff*, R. Ismail, T. S. T. Ismail, K. K. Ng, A. Devi, N. M. Nasir, M. M. Yasin, M. Miskan, E. A. Rahman, M. K. M. Arsad, F. Ariffin, S. A. Razak, F. A. Majid, N. A. Bakar, M. Y. Yacob, N. Zainon, R. Salleh, M. K. A. Ramli, N. A. Halim, S. R. Norlizan, N. M. Ghazali, M. N. Arshad, R. Razali, S. Ali, H. R. Othman, C. W. J. C. W. Hafar, A. Pit, N. Danuri, F. Basir, S. N. A. Zahari, H. Abdullah, M. A. Arippin, N. A. Zakaria, I. Noorhassim, M. J. Hasni, M. T. Azmi, M. I. Zaleha, K. Y. Hazdi, A. R. Rizam, W. Sazman, A. Azman; **Occupied Palestinian Territory:** R. Khatib*, U. Khammash, R. Giacaman; **Pakistan:** R. Iqbal*, R. Khawaja, I. Azam, K. Kazmi; **Peru:** J. Miranda*, A. Bernabe Ortiz, W. Checkley, R. H. Gilman, L. Smeeth, R. M. Carrillo, M. de los Angeles, C. Tarazona Meza; **Philippines:** A. Dans*, H. U. Co, J. T. Sanchez, L. Pudol, C. Zamora-Pudol, L. A. M. Palileo-Villanueva, M. R. Aquino, C. Abaquin, S. L. Pudol, K. Manguiat, S. Malayang; **Poland:** W. Zatonski*, A. Szuba, K. Zatonska, R. Ilow[#], M. Ferus, B. Regulska-Ilow, D. Różańska, M. Wolyniec; **Saudi Arabia:** K. F. AlHabib*, M. Alshamiri, H. B. Altaradi, O. Alnobani, N. Alkamel, M. Ali, M. Abdulrahman, R. Nouri; **South Africa:** L. Kruger*, A. Kruger[#], P. Bestra, H. H. Voster, A. E. Schutte, E. Wentzel-Viljoen, F. C. Eloff, H. de Ridder, H. Moss, J. Potgieter, A. A. Roux, M. Watson, G. de Wet, A. Olckers, J. C. Jerling, M. Pieters, T. Hoekstra, T. Puoane, R. Swart*, E. Igumbor, L. Tsolekile, K. Ndayi, D. Sanders, P. Naidoo, N. Steyn, N. Peer, B. Mayosi[#], B. Rayner, V. Lambert, N. Levitt, T. Kolbe-Alexander, L. Ntyintyane, G. Hughes, J. Fourie, M. Muzigaba, S. Xapa, N. Gobile, K. Ndayi, B. Jwili, K. Ndibaza, B. Egbujie; **Sweden** A. Rosengren*, K. Bengtsson Boström, A. Rawshani, A. Gustavsson, M. Andreasson, L. Wirdemann; **Tanzania:** K. Yeates*, M. Oresto, N. West; **Turkey:** A. Oguz*, N. Imeryuz, Y. Altuntas, S. Gulec, A. Temizhan, K. Karsidag, K. B. T. Calik, A. K. Akalin, O. T. Caklili, M. V. Keskinler, K. Yildiz; **United Arab Emirates:** A. H. Yusufali, F. Hussain, M. H. S. Abdelmotagali, D. F. Youssef, O. Z. S. Ahmad, F. H. M. Hashem, T. M. Mamdouh, F. M. AbdRabbou, S. H. Ahmed, M. A. AlOmairi, H. M. Swidan, M. Omran, N. A. Monsef; **Zimbabwe:** J. Chifamba*, T. Ncube, B. Ncube, C. Chimhete, G. K. Neya, T. Manenji, L. Gwaunza, V. Mapara, G. Terera, C. Mahachi, P. Murambiwa, R. Mapanga, A. Chinhara

*National Coordinator

[#]Deceased

© 2021 Tse LA et al. *JAMA Network Open.*
